# Complete mitochondrial genome of the giant liver fluke *Fascioloides magna* (Digenea: Fasciolidae) and its comparison with selected trematodes

**DOI:** 10.1186/s13071-016-1699-7

**Published:** 2016-08-04

**Authors:** Jun Ma, Jun-Jun He, Guo-Hua Liu, Roman Leontovyč, Martin Kašný, Xing-Quan Zhu

**Affiliations:** 1 State Key Laboratory of Veterinary Etiological Biology, Key Laboratory of Veterinary Parasitology of Gansu Province, Lanzhou Veterinary Research Institute, Chinese Academy of Agricultural Sciences, Lanzhou, Gansu Province 730046 PR China; 2 College of Veterinary Medicine, Hunan Agricultural University, Changsha, Hunan Province 410128 PR China; 3 Department of Parasitology, Faculty of Science, Charles University, Viničná 7, Prague 2, 128 44 Czech Republic; 4 Department of Botany and Zoology, Faculty of Science, Masaryk University, Kotlářská 2, 611 37 Brno, Czech Republic

**Keywords:** *Fascioloides magna*, *Fasciola*, Mitochondrial genome, Phylogenetic analysis

## Abstract

**Background:**

Representatives of the trematode family Fasciolidae are responsible for major socio-economic losses worldwide. *Fascioloides magna* is an important pathogenic liver fluke of wild and domestic ungulates. To date, only a limited number of studies concerning the molecular biology of *F. magna* exist. Therefore, the objective of the present study was to determine the complete mitochondrial (mt) genome sequence of *F. magna*, and assess the phylogenetic relationships of this fluke with other trematodes based on the mtDNA dataset.

**Findings:**

The complete *F. magna* mt genome sequence is 14,047 bp. The gene content and arrangement of the *F. magna* mt genome is similar to those of *Fasciola* spp*.*, except that *trn*E is located between *trn*G and the only non-coding region in *F. magna* mt genome. Phylogenetic relationships of *F. magna* with selected trematodes using Bayesian inference (BI) was reconstructed based on the concatenated amino acid sequences for 12 protein-coding genes, which confirmed that the genus *Fascioloides* is closely related to the genus *Fasciola*; the intergeneric differences of amino acid composition between the genera *Fascioloides* and *Fasciola* ranged 17.97–18.24 %.

**Conclusions:**

The determination of *F. magna* mt genome sequence provides a valuable resource for further investigations of the phylogeny of the family Fasciolidae and other trematodes, and represents a useful platform for designing appropriate molecular markers.

**Electronic supplementary material:**

The online version of this article (doi:10.1186/s13071-016-1699-7) contains supplementary material, which is available to authorized users.

## Background

*Fascioloides magna* (Bassi, 1875), the type- and only species of the genus *Fascioloides* Ward, 1917, was first described as *Distomum magnum* in 1875 [1]. Latter in 1917, Ward erected the genus *Fascioloides* for *Fasciola magna* (Bassi, 1875) [2]. *Fascioloides magna*, known as the large American liver fluke, giant liver fluke or deer fluke, is an important digenetic trematode of the family Fasciolidae [3, 4]. This species, which is of North America origin [5, 6] and invasive in European countries [7], has high potential to colonize new geographic territories (a variety of wild and domestic ungulates [3, 8–10]), and can establish expanding populations from a natural epidemic focus through translocated hosts [5, 6, 11]. Migration of *F. magna* immature flukes within the host body often leads to profound damage to the liver and other organ tissues [8, 12], causing economic losses worldwide [13].

The consequences of infection of various intermediate and definitive hosts by *F. magna* has been intensively studied [8, 12], but the relevant molecular research of this fluke has not received enough attention [4, 9]. To date, a sequence of nuclear ribosomal DNA (rDNA) of *F. magna* was obtained in 2008 [14], partial sequences of mitochondrial (mt) genes, such as cytochrome *c* oxidase subunit I (*cox*1) and nicotinamide dehydrogenase subunit I (*nad*1) were characterized [3]. According to these data, *F. magna* was divided into two mt haplotype groups [5, 14, 15], the first haplotype representing isolates from western North America and Italy, and the second haplotype representing isolates from eastern North America and some European countries such as Czech Republic, Poland and Croatia [3, 5]. Recently, the *F. magna* transcriptome was reported, which provides a useful platform for further fundamental studies of this fluke [16], but complete mt genome of *F. magna* is still unavailable.

Molecular tools, using genetic markers in mitochondrial DNA (mtDNA) sequences, have been proven reliable in identification and differentiation of trematode species [17–20]. In the present study we determined the mitochondrial genome sequence of *F. magna* (Czech isolate) using PCR-coupled sequencing technique combined with bioinformatic analysis, and for the first time assessed its phylogenetic relationship with selected trematodes based on the nucleotide- and inferred amino acid sequences of the protein-coding genes.

## Methods

### Sampling and DNA extraction

Three adult *F. magna* worms were isolated from livers of naturally infected red deer (*Cervus elaphus*), hunted at Kokořínsko area, Czech Republic. Worms were washed in 0.1 M phosphate-buffered saline (PBS), pH 7.2, fixed in 70 % (v/v) ethanol and preserved at -20 °C, until further use. Total genomic DNA was extracted from individual *F. magna* specimens using sodium dodecyl sulfate (SDS)/proteinase K treatment [21] and column-purification (Wizard® SV Genomic DNA Purification System, Promega, Madison, USA), according to the manufacturer’s protocol.

### Acquisition of ITS rDNA and sample identification

The internal transcribed spacer (ITS) rDNA region of each of the three *F. magna* specimens, spanning partial 18S rDNA, the complete ITS-1, 5.8S rDNA, ITS-2, and partial 28S rDNA, was amplified using primers BD1 (forward; 5’-GTC GTA ACA AGG TTT CCG TA-3’ and BD2 (reverse; 5’-ATG CTT AAA TTC AGC GGG T-3’) [22] and sequenced using the same primers. These *F. magna* samples had ITS-1 and ITS-2 sequences identical to the corresponding sequences available on GenBank (EF051080).

### Long-range PCR-based sequencing of mt genome

The primers were designed based on relatively conserved regions of mtDNA sequences from *Fasciola hepatica* and *Fasciola gigantica*. The entire mt genome from a single specimen of *F. magna* was amplified in 5 overlapping fragments, using the primers shown in Additional file 1: Table S1.

PCR reactions were conducted in a total volume of 50 μl, using 25 μl PrimeStar Max DNA polymerase premix (Takara, Dalian, China), 25 pmol of each primer (synthesized in Genewiz, Suzhou, China), 0.5 μl DNA templates, and H_2_O, in a thermocycler (Biometra, Göttingen, Germany). PCR cycling conditions started with an initial denaturation at 98 °C for 2 min, followed by 22 cycles of denaturation at 92 °C for 18 s, annealing at 52–65 °C for 12 s and extension at 60 °C for 1–5 min, followed by 92 °C denaturation for 2 min, plus 25 cycles of 92 °C for 18 s (denaturation), 50–67 °C for 12 s (annealing) and 66 °C for 3–6 min, with a final extension step for 10 min at 66 °C. A negative control (no DNA) was included in each amplification run. Amplicons (2.5 μl) were electrophoresed in a 2 % agarose gel, stained with Gold View I (Solarbio, Beijing, China) and photographed by GelDoc - It TS™ Imaging System (UVP, USA).

### Assembly, annotation and bioinformatics analysis

Sequences were assembled manually and aligned against the entire mt genome sequences of *Fa. hepatica* (GenBank accession No. NC_002546) and *Fa. gigantica* (NC_024025) using MAFFT 7.122 to infer boundaries for each gene. Amino acid sequences of 12 protein-coding genes were translated using MEGA v.6.06 and NCBI translation Table 21 (Trematode Mitochondrial Code). The tRNA genes were affirmed using the programs tRNAscan-SE [23] and ARWEN (http://130.235.46.10/ARWEN/) or by comparison with those from the *Fa. hepatica* and *Fa. gigantica* mt genomes. The two rRNA genes were identified by comparison with those of *Fa. hepatica* and *Fa. gigantica*.

A comparative analysis of the nucleotide sequences of each protein-coding gene, the amino acid sequences, two ribosomal RNA genes, 22 tRNA genes as well as non-coding regions (NCRs) among *F. magna*, *Fa. hepatica* and *Fa. gigantica* was conducted.

### Phylogenetic analysis

The concatenated amino acid sequences of *F. magna* mt genome, conceptually translated from individual genes of each mt genome, were aligned with those of published mt genomes from selected trematodes, including *Opisthorchis felineus* (GenBank acession No. EU_921260) and *Clonorchis sinensi*s (FJ_381664) (Opisthorchiidae); *Metagonimus yokogawai* (KC_330755) and *Haplorchis taichui* (KF_214770) (Heterophyidae); *Paragonimus westermani* Japanese isolate (AF219379) and *Paragonimus westermani* Indian isolate (NC_027673) (Paragonimidae); *Fa. hepatica*, *Fasciola* sp. (KF_543343) and *Fa. gigantica* (Fasciolidae); *Hypoderaeum* sp. (KM111525) (Echinostomatidae); *Paramphistomum leydeni* (KP341657) and *Fischoederius elongatus* (KM397348) (Paramphistomatidae); *Diplostomum spathaceum* (KR269763) and *Diplostomum pseudospathaceum* (KR269764) (Diplostomidae); *Ogmocotyle sikae* (KR006934) (Notocotylidae); *Eurytrema pancreaticum* (KP241855) (Dicrocoeliidae); *Schistosoma turkestanicum* (HQ_283100) and *Schistosoma japonicum* (HM_120842) (Schistosomatidae). The sequence for the monogenean *Gyrodactylus derjavinoides* (NC_010976) (Gyrodactylidae), was included as the outgroup.

All inferred amino acid sequences were aligned using MAFFT 7.122. Poorly aligned sites and divergent regions of the alignment were eliminated using Gblocks Server v. 0.91b (http://molevol.cmima.csic.es/castresana/Gblocks_server.html) using default settings, selecting the option of less strict conservation of flanking positions. The alignment was then converted into nexus format using Clustal X1.83 and subjected to phylogenetic analysis using Bayesian inference (BI). A mixed model was used in BI analysis using MrBayes 3.1.1 [24], because the most suitable amino acid evolution model JTT + G + F, selected by ProTest 3.4 based on the Akaike information criterion (AIC) [25], was not available in the current MrBayes version. Four independent Markov chain were run for 10,000,000 metropolis-coupled MCMC generations, sampling trees every 1,000 generations. The first 2,500 trees (25 %) were discarded as ‘burn-in’, and the remaining trees were used for calculating Bayesian posterior probabilities. The analysis was regarded as completed when the potential scale reduction factor was close to 1, and the average standard deviation of split frequencies was below 0.01. Phylograms were prepared using FigTree v. 1.42 [26].

## Findings

### Genome content and organization

**Fig. 1:**
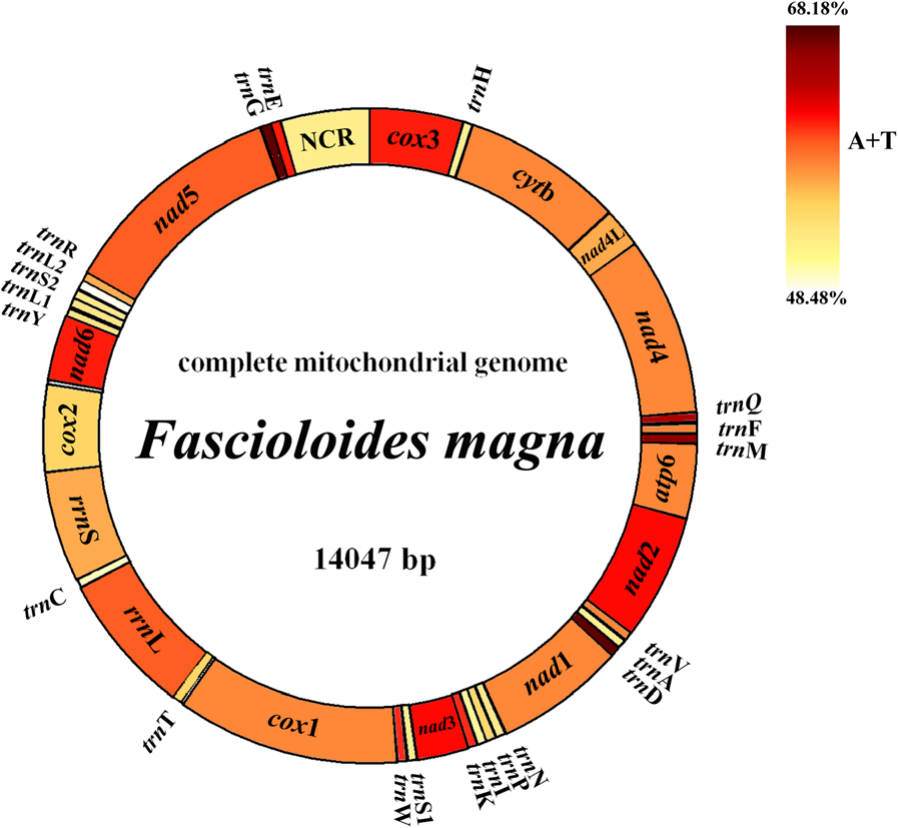
Organization of the mitochondrial genome of *Fascioloides magna*. The scales are approximate. All genes are transcribed in the clockwise direction, using standard nomenclature. “NCR” refers to the only non-coding region in *F. magna* data. The A + T content is shown for each gene or region of the mt genome and represented by colour

The complete mt genome sequence of *F. magna* (GenBank accession no. KR006934) is 14,047 bp in length (Fig. 1) and contains 36 genes that are transcribed in the same direction, including 12 protein-coding genes (*nad*1-6, *nad*4L, *cox*1-3, *atp*6 and *cyt*b), 22 tRNA genes and two rRNA genes (*rrn*L and *rrn*S), lacking the *atp*8 gene (Table 1), consistent with those of selected trematode species available on GenBank [17–19, 27, 28]. There is only one NCR in *F. magna* mt genome, whereas the mt genomes of *Fasciola* flukes have two non-coding regions [17, 27].

**Table 1:** The features of the mitochondrial genomes of *Fascioloides magna*

Gene	Coding position (5’–3’)	Length (bp)	Start/Stop codons	No. of amino acids	Intergenic nucleotides
*cox*3	1–645	645	ATG/TAA	215	4
*trn*H	650–713	64			1
*cyt*b	715–1827	1113	ATG/TAG	371	7
*nad*4L	1835–2107	273	GTG/TAG	91	-40
*nad*4	2068–3348	1281	GTG/TAG	427	1
*trn*Q	3350–3412	63			11
*trn*F	3424–3486	63			14
*trn*M	3501–3566	66			0
*atp*6	3567–4085	516	ATG/TAA	172	4
*nad*2	4090–4959	870	ATG/TAG	290	2
*trn*V	4962–5023	62			7
*trn*A	5031–5092	62			6
*trn*D	5099–5160	62			1
*nad*1	5162–6064	903	GTG/TAG	301	7
*trn*N	6072–6137	66			4
*trn*P	6142–6210	69			0
*trn*I	6211–6273	63			5
*trn*K	6279–6343	65			0
*nad*3	6344–6700	357	ATG/TAA	119	4
*trn*S1	6705–6763	59			8
*trn*W	6772–6836	65			3
*cox*1	6840–8384	1545	GTG/TAG	515	23
*trn* T	8408–8469	62			0
*rrn*L	8470–9453	984			2
*trn*C	9456–9518	63			-2
*rrn*S	9517–10281	765			2
*cox*2	10284–10886	603	ATG/TAG	201	32
*nad*6	10919–11371	453	GTG/TAG	151	0
*trn*Y	11372–11428	57			12
*trn*L1	11441–11504	64			2
*trn*S2	11506–11566	60			10
*trn*L2	11577–11642	66			-3
*trn*R	11640–11705	66			-2
*nad*5	11704–13272	1569	GTG/TAG	523	10
*trn*G	13283–13348	66			6
*trn*E	13355–13422	68			0
NCR	13423–14047	520			0

*Abbreviation*: *NCR* Non-coding region

The arrangement of genes in the *F. magna* mt genome is similar to that of *Fasciola* spp. [17], except that only one non-coding region (NCR) in *F. magna* mt genome is located between *trn*E (13,355–13,422) and *cox*3 (1–645) (Table 1). The gene order of *F. magna* mt genes is similar to that in species of the Paramphistomatidae, Notocotylidae, Echinostomatidae, Heterophyidae and Opisthorchiidae, but is distinct from some flukes of the Schistosomatidae (*S. mansoni*, *S. spindale* and *S. haematobium*) [29].

The nucleotide composition of *F. magna* mt genome is obviously biased towards A and T. The value of total A + T content for *F. magna* mtDNA is 61.42 %, within the range recognized in other trematode mt genomes (54.38 % in *Paragonimus westermani* Indian isolates [30], 72.71 % in *Schistosoma spindale* [29]). The content of C is low (10.3 %) and that of T is high (44.0 %). The A + T content for each gene or region of *F. magna* mt genome ranged from 48.48 % (*trn*L2) to 68.18 % (*trn*G) (*nad*3, 64.43 %; *cox*2, 59.7 %). All 12 protein-coding genes of *F. magna* mtDNA possess a lower A + T percentage than those of *Fa. hepatica* and *Fa. gigantica* [17, 27], except for *nad*5 (Additional file 2: Table S2).

### Annotation of *F. magna* mt genome

In the mt genome of *F. magna*, the protein-coding genes had ATG or GTG as start codons and TAG or TAA as stop codons (Table 1). Half of the protein-coding genes of *F. magna* were initiated with GTG (*nad*4L, *nad*4, *nad*1, *cox*1, *nad*6 and *nad*5). Incomplete codons were not detected in the mt genome of *F. magna*.

The 22 tRNA genes of *F. magna* mt genome ranged from 57 to 69 bp in length. The structure of all tRNA sequences is similar to those of *Fa. hepatica* and *Fa. gigantica* [17, 27]. The large ribosomal RNA gene (*rrn*L) and the adjacent small ribosomal RNA gene (*rrn*S) are located between *trn*T and *cox*2, and separated by *trn*C (9,456–9,518) (Table 1). The length of the *rrn*L and *rrn*S RNA genes is 984 bp and 765 bp, respectively. The only NCR of *F. magna* mt genome is of 520 bp in length, and is located between *trn*E and *cox*3. It contains two complete direct repeats: six copies of a 23 nt - repeat A (AGA TAG GAT AGG CAT CTG GTA TA) and five copies of a 37 nt - repeat B (GGT GCC CCC GGT GAA GGG GGA AAA GGA AGG TTG TAA G). There are five AB repeats, with one A at the end (located at positions 13,620–13,642).

### Comparative analysis among mt genomes of *F. magna*, *Fa. hepatica* and *Fa. gigantica*

The difference between complete mt genomes of *F. magna* and *Fa. hepatica* was 22.66 % (3,290 nt), which is close to that between *F. magna* and *Fa. gigantica* (22.65 %, 3,297 nt) (Table 2). Considering the 12 protein-coding genes, different nucleotides were present at 18.80 % of positions (1,897 nt) between *F. magna* and *Fa. hepatica*, and at 18.62 % of positions (1,879 nt) between *F. magna* and *Fa. gigantica*. At the inferred amino acid level, there were 605 substitutions (17.97 %) of amino acids between *F. magna* and *Fa. hepatica*, and 614 substitutions (18.24 %) between *F. magna* and *Fa. gigantica* (Table 2).

**Table 2:** Comparison of nucleotides and predicted amino acids sequences among *Fascioloides magna* (Fm), *Fasciola hepatica* (Fh) and *Fasciola gigantica* (Fg)

Gene	nt difference (%)			aa difference (%)		
	Fm/Fh	Fm/Fg	Fh/Fg	Fm/Fh	Fm/Fg	Fh/Fg
*cox*3	19.8	18.7	13.4	20.1	22.4	13.1
*cyt*b	16.1	16.4	8.3	11.6	13.2	6.5
*nad*4L	15.3	16.1	8.4	12.1	15.4	5.5
*nad*4	21.4	20.9	13.5	21.7	20.8	10.6
*atp*6	21.3	21.2	13.8	22.0	20.2	13.3
*nad*2	21.1	22.8	11.6	25.4	24.7	11.4
*nad*1	15.0	14.9	8.4	13.0	14.3	6.6
*nad*3	19.0	19.0	10.6	13.4	15.1	7.6
*cox*1	13.1	12.8	9.1	9.2	8.4	5.5
*cox*2	19.2	19.2	11.6	14.4	15.4	6.5
*nad*6	24.2	26.2	16.3	22.5	27.8	13.2
*nad*5	21.5	19.8	13.7	23.6	22.8	12.3
*rrn*L	18.3	16.6	10.6			
*rrn*S	22.2	21.4	11.1			
22 tRNAs	16.3	16.0	9.9			
Overall	22.7	22.7	12.2	17.97	18.24	9.4

At the nucleotide level, sequence differences in protein-coding genes ranged from 13.1 to 24.2 % (between *F. magna* and *Fa. hepatica*) and from 12.8 to 26.2 % (between *F. magna* and *Fa. gigantica*), with *cox*1, *nad*1, *nad*4L and *cyt*b being the most conserved genes, and *nad*6, *nad*5 and *nad*2 being the least conserved genes among those three species. At the amino acid level, sequence differences ranged from 9.2 to 25.4 % between *F. magna* and *Fa. hepatica*, and from 8.4 to 27.8 % between *F. magna* and *Fa. gigantica*: *cox*1, *cyt*b, *nad*4L and *nad*1 were the most conserved protein-coding genes, while *nad*6, *nad*2 and *nad*5 were the least conserved.

Comparisons between the mt genomes of *F. magna* and *Fasciola* spp., at both nucleotide and amino acid levels, indicate that the most conserved and the least conserved gene in the Fasciolidae are *cox*1 and *nad*6, respectively. Besides, the *nad*5 is highly variable, and genes of *nad*4L and *cyt*b are rather conserved. These characteristics are in accordance with flukes of the families Paramphistomatidae and Notocotylidae [18, 28].

Nucleotide differences were also found in ribosomal RNA genes: between *F. magna* and *Fa. hepatica* (*rrn*L, 18.3 %; *rrn*S, 22.2 %) and between *F. magna* and *Fa. gigantica* (*rrn*L, 16.6 %; *rrn*S, 21.4 %) as well as in tRNA genes (16.3 % between *F. magna* and *Fa. hepatica* and 16.0 % between *F. magna* and *Fa. gigantica*). Meaningful sequence comparisons of NCRs in mt genomes of the three fasciolid trematodes is not possible, because there is only one NCR present in *F. magna* mt genome, while in both *Fa. hepatica* and *Fa. gigantica* there are two NCRs.

### Phylogenetic analysis

**Fig. 2:**
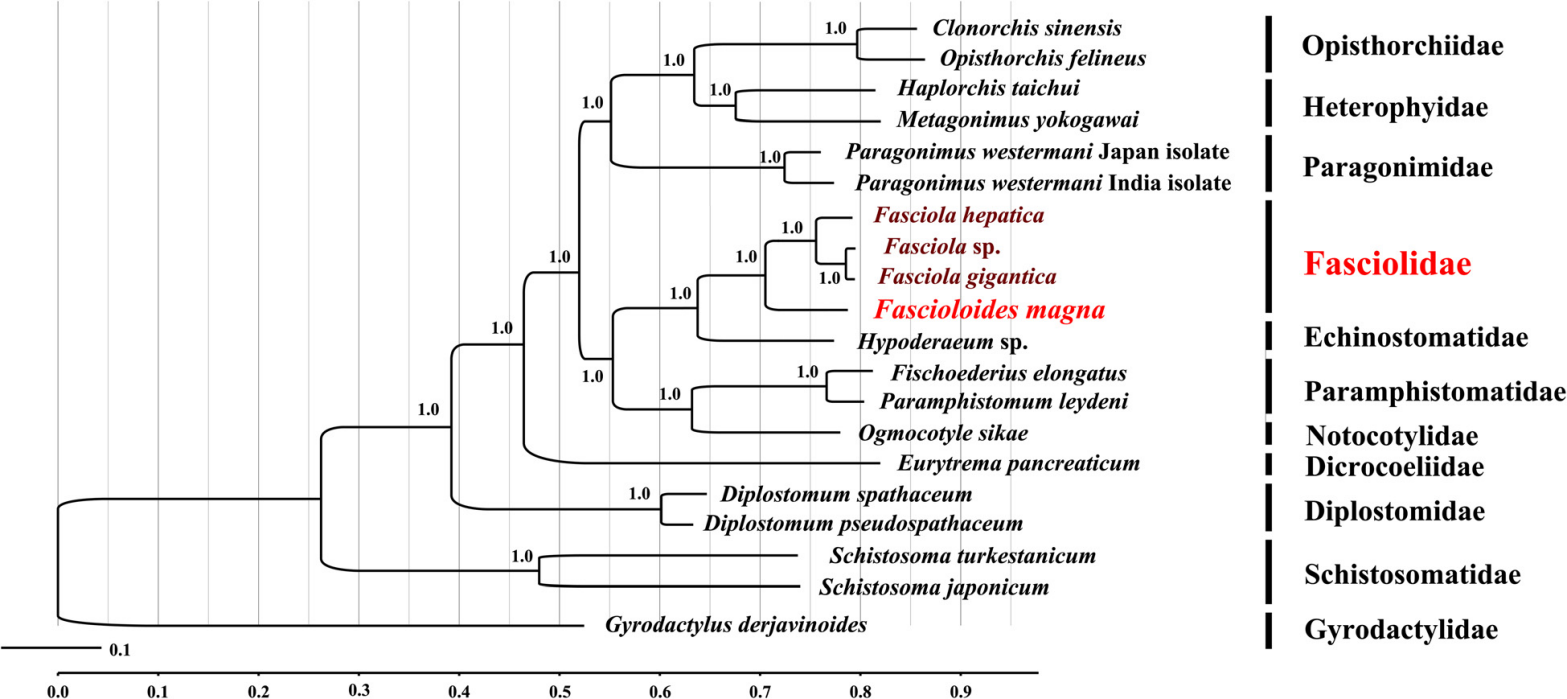
Phylogenetic relationships of *Fascioloides magna* and other trematodes. Tree inferred from the concatenated amino acid sequence dataset for 12 protein-coding genes from 19 trematodes was performed by Bayesian inference (BI). *Gyrodactylus derjavinoides* (NC_010976) was chosen as the outgroup

In the phylogenic tree inferred from the concatenated amino acid sequence dataset of all 12 mt proteins (Fig. 2) *F. magna* clustered with three other *Fasciola* species with strong support (Bpp = 1). The closest family to the Fasciolidae is Echinostomatidae, represented by *Hypoderaeum* sp. The taxonomic relationships of the selected trematodes are in concordance with results of previous studies [17–19, 28]. Each node received the maximum possible nodal support (Bpp = 1).

In several recent phylogenetic studies, the *F. magna* was characterized only based on partial 28S rDNA [31] and combined ITS1, ITS2 and *nad*1 sequences [32]. The relationship between the genera *Fasciola* and *Fasciolopsis* was considered as being very close and the genetic relationship between *F. magna* and *Fasciola jacksoni* (or *Fascioloides jacksoni*) is disputable [31–33]. Further studies are warranted to determine the mt genome of *Fa. jacksoni* and solve this controversy in the family Fasciolidae.

## Conclusions

The present study determined the complete mt genome sequence of the pathogenic liver fluke *F. magna* and revealed its close relationship with the species of *Fasciola*. The complete mt genome data of *F. magna* provides a resource for further investigations of the phylogeny, epidemiology, biology and population genetics of the family Fasciolidae and other trematodes.

## Abbreviations

mt, mitochondrial; mtDNA, mitochondrial DNA; rDNA, ribosomal DNA; BI, Bayesian inference; PBS, phosphate-buffered saline; SDS, sodium dodecyl sulphate; ITS, internal transcribed spacer; NCR, non-coding region

## Additional files

Additional file 1: Table S1. Sequences of primers used to amplify fragments of *Fascioloides magna* mitochondrial genome. (DOCX 13 kb) Additional file 2: Table S2. Comparison of A + T content of mitochondrial genomes of *Fascioloides magna* (Fm), *Fasciola hepatica* (Fh) and *Fasciola gigantica* (Fg). (DOCX 20 kb)
