# Correction: Complete mitochondrial genome of the giant liver fluke *Fascioloides magna* (Digenea: Fasciolidae) and its comparison with selected trematodes

**DOI:** 10.1186/s13071-026-07577-w

**Published:** 2026-09-10

**Authors:** Jun Ma, Jun-Jun He, Guo-Hua Liu, Roman Leontovyč, Martin Kašný, Xing-Quan Zhu

**Affiliations:** 1 State Key Laboratory of Veterinary Etiological Biology, Key Laboratory of Veterinary Parasitology of Gansu Province, Lanzhou Veterinary Research Institute, Chinese Academy of Agricultural Sciences, Lanzhou, 730046 Gansu People’s Republic of China; 2 College of Veterinary Medicine, Hunan Agricultural University, Changsha, Hunan 410128 People’s Republic of China; 3 Department of Parasitology, Faculty of Science, Charles University, Viničná 7, Prague 2, 128 44 Czech Republic; 4 Department of Botany and Zoology, Faculty of Science, Masaryk University, Kotlářská 2, 611 37 Brno, Czech Republic

**Correction: Parasites & Vectors (2016) 9:429** **https://doi.org/10.1186/s13071-016-1699-7**

Following publication of the original article [1] it was brought to the journal’s attention by a reader that the GenBank accession no. of *F. magna* had been incorrectly reported: it had been reported as KR006934 rather than KU060148, the correct number. The authors thank you for reading this erratum and apologize for any inconvenience caused.

## Data Availability

The datasets supporting the conclusions of this article are included within the article and its additional files. The complete mt genome sequence of *F. magna* is deposited in the GenBank database under the accession number KU060148.
