# Supplementary material for: Complete mitochondrial genome of the giant liver fluke Fascioloides magna (Digenea: Fasciolidae) and its comparison with selected trematodes
Source: Parasit Vectors. 2016 Aug 4;9:429. doi: 10.1186/s13071-016-1699-7 (PMC4973546; doi:10.1186/s13071-016-1699-7)
Supplement: Additional file 1: Table S1. — Sequences of primers used to amplify fragments of Fascioloides magna mitochondrial genome. (DOCX 13 kb) [file 13071_2016_1699_MOESM1_ESM.docx]

**Additional file 1**

**Table S1** Sequences of primers used to amplify fragments of *Fascioloides magna*mitochondrial genome

| **Primer** | **Sequence (5′–3′)** | **Size (kb)** | **Amplified region** |
| --- | --- | --- | --- |
| Fm1F | TCTATATTAGGTTCTGGGAGTGTG | ~3 | Partial *nad*5 - partial *cyt*b |
| Fm1R | GGCTTTATAGACACAGGAGTCACCATAG |  |  |
| Fm2F | AGATGTGGAGAGTTATATTCAGGC | ~0.6 | Partial *cyt*b - partial *nad*4L |
| Fm2R | CTAAACACCAATTATACCAATCAAA |  |  |
| Fm3F | GTGTTGTTGAGAATCTTAATGTGTTGT | ~5.4 | Partial *nad*4L - partial *nad*1 |
| Fm3R | ACACAAACTTAAACTTTATGACCAACTTCAA |  |  |
| Fm4F | GCAGGTTCGTAAGGGGCCTAATA | ~4.9 | Partial *nad*1 - partial *rrn*S |
| Fm4R | CCAGCATTACCATGTTACGACTT |  |  |
| Fm5F | CAGGACTCGGAAGTAGGCGGATT | ~3 | Partial *rrn*S - partial *nad*5 |
| Fm5R | CCCCTCAAAGAACAGAACTCCAA |  |  |
