# Supplementary material for: Complete mitochondrial genome of the giant liver fluke Fascioloides magna (Digenea: Fasciolidae) and its comparison with selected trematodes
Source: Parasit Vectors. 2016 Aug 4;9:429. doi: 10.1186/s13071-016-1699-7 (PMC4973546; doi:10.1186/s13071-016-1699-7)
Supplement: Additional file 2: Table S2. — Comparison of A + T content of mitochondrial genomes of Fascioloides magna (Fm), Fasciola hepatica (Fh) and Fasciola gigantica (Fg). (DOCX 20 kb) [file 13071_2016_1699_MOESM2_ESM.docx]

**Additional file 2**

**Table S2** Comparison of A+T content of mitochondrial genomes of [*Fascioloides magna*](http://blast.ncbi.nlm.nih.gov/Blast.cgi#alnHdr_685426884) (Fm), [*Fasciola hepatica*](http://blast.ncbi.nlm.nih.gov/Blast.cgi#alnHdr_685426884) (Fh) and [*Fasciola gigantica*](http://blast.ncbi.nlm.nih.gov/Blast.cgi#alnHdr_685426884) (Fg)

| **Gene** | **A (%)** | | | **G (%)** | | | **T (%)** | | | **C (%)** | | | **A+T (%)** | | |
| --- | --- | --- | --- | --- | --- | --- | --- | --- | --- | --- | --- | --- | --- | --- | --- |
|  | **Fm** | **Fh** | **Fg** | **Fm** | **Fh** | **Fg** | **Fm** | **Fh** | **Fg** | **Fm** | **Fh** | **Fg** | **Fm** | **Fh** | **Fg** |
| *cox*3 | 16.7 | 16.0 | 15.4 | 26.5 | 23.8 | 23.7 | 47.0 | 48.8 | 52.3 | 9.8 | 11.4 | 8.6 | 63.7 | 64.8 | 67.8 |
| *trn*H | 20.3 | 18.8 | 18.8 | 34.4 | 35.9 | 34.4 | 34.4 | 32.8 | 35.9 | 10.9 | 12.5 | 10.9 | 54.7 | 51.6 | 54.7 |
| *cyt*b | 16.4 | 14.7 | 14.3 | 28.1 | 27.9 | 28.1 | 45.1 | 47.4 | 48.6 | 10.4 | 10.1 | 9.0 | 61.5 | 62.1 | 62.9 |
| *nad*4L | 15.8 | 14.3 | 13.6 | 31.9 | 28.2 | 29.7 | 45.1 | 51.7 | 51.7 | 7.3 | 5.9 | 5.1 | 60.8 | 65.9 | 65.2 |
| *nad*4 | 14.9 | 14.0 | 12.7 | 28.5 | 27.7 | 28.4 | 46.8 | 48.7 | 50.1 | 9.8 | 9.6 | 8.8 | 61.7 | 62.7 | 62.8 |
| *trn*Q | 23.8 | 19.1 | 18.2 | 27.0 | 33.3 | 31.8 | 42.9 | 39.7 | 42.4 | 6.4 | 7.9 | 7.6 | 66.7 | 58.7 | 60.6 |
| *trn*F | 30.2 | 28.8 | 26.2 | 25.4 | 27.3 | 27.7 | 31.8 | 31.8 | 33.9 | 12.7 | 12.1 | 12.3 | 61.9 | 60.6 | 60.0 |
| *trn*M | 31.8 | 27.5 | 28.8 | 19.7 | 23.2 | 19.7 | 33.3 | 37.7 | 37.9 | 15.2 | 11.6 | 13.6 | 65.2 | 65.2 | 66.7 |
| *atp*6 | 15.2 | 12.3 | 11.2 | 26.8 | 25.6 | 27.8 | 45.9 | 51.1 | 52.0 | 12.1 | 11.0 | 9.1 | 61.1 | 63.4 | 63.2 |
| *nad*2 | 13.2 | 12.6 | 12.5 | 26.8 | 25.4 | 25.1 | 50.8 | 53.4 | 53.8 | 9.2 | 8.7 | 8.7 | 64.0 | 66.0 | 66.2 |
| *trn*V | 24.2 | 20.6 | 23.4 | 25.8 | 27.0 | 21.9 | 37.1 | 41.3 | 42.2 | 12.9 | 11.1 | 12.5 | 61.3 | 61.9 | 65.6 |
| *trn*A | 21.0 | 17.5 | 16.9 | 30.7 | 33.3 | 30.8 | 33.9 | 34.9 | 38.5 | 14.5 | 14.3 | 13.9 | 54.8 | 52.4 | 55.4 |
| *trn*D | 24.2 | 15.2 | 16.9 | 22.6 | 34.9 | 29.2 | 43.6 | 39.4 | 43.1 | 9.7 | 10.6 | 10.8 | 67.7 | 54.6 | 60.0 |
| *nad*1 | 14.6 | 15.0 | 13.2 | 30.8 | 28.1 | 29.5 | 46.6 | 50.1 | 50.2 | 8.0 | 6.9 | 7.2 | 61.2 | 65.0 | 63.3 |
| *trn*N | 25.8 | 21.4 | 22.9 | 30.3 | 31.4 | 24.3 | 30.3 | 34.3 | 38.6 | 13.6 | 12.9 | 14.3 | 56.1 | 55.7 | 61.4 |
| *trn*P | 17.4 | 17.9 | 22.1 | 30.4 | 37.3 | 30.9 | 42.0 | 35.8 | 38.2 | 10.1 | 9.0 | 8.8 | 59.4 | 53.7 | 60.3 |
| *trn*I | 17.5 | 19.4 | 17.7 | 31.8 | 32.3 | 32.3 | 36.5 | 33.9 | 35.5 | 14.3 | 14.5 | 14.5 | 54.0 | 53.2 | 53.2 |
| *trn*K | 23.1 | 23.9 | 20.9 | 23.1 | 25.4 | 25.4 | 40.0 | 38.8 | 41.8 | 13.9 | 11.9 | 11.9 | 63.1 | 62.7 | 62.7 |
| *nad*3 | 13.2 | 13.7 | 12.9 | 29.1 | 25.8 | 26.1 | 51.3 | 52.9 | 52.9 | 6.4 | 7.6 | 8.1 | 64.4 | 66.7 | 65.8 |
| *trn*S1 | 15.3 | 12.1 | 12.5 | 30.5 | 32.8 | 28.6 | 40.7 | 39.7 | 42.9 | 13.6 | 15.5 | 16.1 | 55.9 | 51.7 | 55.4 |
| *trn*W | 24.6 | 17.5 | 20.6 | 29.2 | 31.8 | 28.6 | 38.5 | 38.1 | 39.7 | 7.7 | 12.7 | 11.1 | 63.1 | 55.6 | 60.3 |
| *cox*1 | 16.5 | 15.1 | 14.3 | 27.1 | 26.0 | 25.7 | 45.1 | 47.9 | 49.2 | 11.3 | 11.0 | 10.8 | 61.6 | 63.0 | 63.5 |
| *trn*T | 25.8 | 17.4 | 19.1 | 25.8 | 30.4 | 29.4 | 41.9 | 42.0 | 38.2 | 6.5 | 10.1 | 13.2 | 67.7 | 59.4 | 57.4 |
| *rrn*L | 22.4 | 22.4 | 20.6 | 26.4 | 26.1 | 27.8 | 40.1 | 40.7 | 41.4 | 11.1 | 10.7 | 10.2 | 62.5 | 63.1 | 62.0 |
| *trn*C | 15.9 | 19.1 | 16.9 | 25.4 | 25.4 | 29.2 | 34.9 | 38.1 | 35.4 | 23.8 | 17.5 | 18.5 | 50.8 | 57.1 | 52.3 |
| *rrn*S | 24.2 | 24.0 | 22.2 | 26.7 | 26.1 | 26.7 | 36.0 | 37.3 | 38.7 | 13.2 | 12.5 | 12.5 | 60.1 | 61.4 | 60.8 |
| *cox*2 | 22.9 | 21.9 | 21.9 | 27.7 | 26.4 | 25.9 | 36.8 | 40.5 | 42.0 | 12.6 | 11.3 | 10.3 | 59.7 | 62.4 | 63.9 |
| *nad*6 | 14.8 | 13.5 | 13.0 | 24.7 | 24.5 | 26.3 | 48.6 | 51.2 | 51.0 | 11.9 | 10.8 | 9.7 | 63.4 | 64.7 | 64.0 |
| *trn*Y | 19.3 | 17.5 | 17.5 | 33.3 | 33.3 | 35.1 | 38.6 | 40.4 | 36.8 | 8.8 | 8.8 | 10.8 | 57.9 | 57.9 | 54.4 |
| *trn*L1 | 21.9 | 18.2 | 16.9 | 29.7 | 28.8 | 27.7 | 35.9 | 39.4 | 41.5 | 12.5 | 13.6 | 13.9 | 57.8 | 57.6 | 58.5 |
| *trn*S2 | 15.0 | 21.0 | 17.5 | 30.0 | 21.0 | 22.8 | 41.7 | 45.2 | 47.4 | 13.3 | 12.9 | 12.3 | 56.7 | 66.1 | 64.9 |
| *trn*L2 | 16.7 | 19.7 | 20.6 | 36.4 | 28.8 | 30.2 | 31.8 | 36.4 | 33.3 | 15.2 | 15.2 | 15.9 | 48.5 | 56.1 | 54.0 |
| *trn*R | 16.7 | 19.7 | 15.2 | 25.8 | 24.2 | 25.8 | 43.9 | 43.9 | 45.5 | 13.6 | 12.1 | 13.6 | 60.6 | 63.6 | 60.6 |
| *nad*5 | 13.1 | 11.4 | 10.8 | 30.4 | 29.3 | 29.3 | 49.1 | 50.7 | 52.6 | 7.5 | 8.7 | 7.3 | 62.1 | 62.1 | 63.4 |
| *trn*G | 31.8 | 20.6 | 17.2 | 19.7 | 27.0 | 25.0 | 36.4 | 38.1 | 42.2 | 12.1 | 14.3 | 15.6 | 68.2 | 58.7 | 59.4 |
| *trn*E | 16.2 | 18.8 | 17.7 | 30.9 | 24.6 | 25.0 | 47.1 | 42.0 | 47.1 | 5.9 | 14.5 | 10.3 | 63.2 | 60.9 | 64.7 |
| NCR | 25.8 | 17.6 | 18.4 | 33.0 | 41.5 | 39.0 | 29.8 | 32.4 | 34.1 | 11.5 | 8.5 | 8.4 | 55.5 | 50.1 | 52.6 |
| Overall | 17.5 | 16.1 | 15.3 | 28.3 | 27.9 | 28.0 | 44.0 | 46.1 | 47.4 | 10.3 | 9.9 | 9.4 | 61.4 | 62.2 | 62.7 |
